# The impact of MYD88 and PIM1 in mature large B-cell non-Hodgkin lymphomas: Defining element of their evolution and prognosis

**DOI:** 10.1097/MD.0000000000036269

**Published:** 2024-02-09

**Authors:** Miruna Cristian, Mariana Așchie, Anca-Florentina Mitroi, Mariana Deacu, Mădălina Boșoteanu, Gabriela-Izabela Bălțătescu, Andreea-Georgiana Stoica, Anca-Antonela Nicolau, Manuela Enciu, Ana-Maria Crețu, Andreea-Daniela Caloian, Cristian-Ionuț Orășanu, Ionuț Poinăreanu

**Affiliations:** a Faculty of Medicine, “Ovidius” University of Constanta, Constanța, Romania; b Center for Research and Development of the Morphological and Genetic Studies of Malignant Pathology – CEDMOG, “Ovidius” University of Constanta, Constanța, Romania; c Department of Clinical Pathology, “Sf. Apostol Andrei” Emergency County Hospital, Constanta, Romania; d Academy of Medical Sciences, Bucharest, Romania; e Academy of Romanian Scientists, Bucharest, Romania; f Department of Hematology, “Sf. Apostol Andrei” Emergency County Hospital, Constanta, Romania; g Department of Hemato-Oncology, “Ovidius” Clinical Hospital, Constanta, Romania; h Department of Pathology, Săcele Municipal Hospital, Brasov, Romania.

**Keywords:** diffuse large B-cell lymphoma, human myeloid differentiation primary response gene (88) protein, immunocytochemistry, molecular subtype, mutation profiling, pro-to-oncogene protein PIM1

## Abstract

Sequence studies of the entire exome and transcriptome of lymphoma tissues have identified *MYD88* and *PIM1* as involved in the development and oncogenic signaling. We aimed to determine the frequency of *MYD88* and *PIM1* mutations, as well as their expressions in conjunction with the clinicopathological parameters identified in mature large B-cell non-Hodgkin lymphomas. The ten-year retrospective study included 50 cases of mature large B-cell lymphoma, diagnosed at the Pathology Department of the Emergency County Hospital of Constanţa and Săcele County Hospital of Brasov. They were statistically analyzed by demographic, clinicopathological, and morphogenetic characteristics. We used a real-time polymerase chain reaction technique to identify *PIM1* and *MYD88* mutations as well as an immunohistochemical technique to evaluate the expressions of the 2 genes. Patients with lymphoma in the small bowel, spleen, brain, and testis had a low-performance status Eastern Cooperative Oncology Group (*P* = .001). The Eastern Cooperative Oncology Group performance status represented an independent risk factor predicting mortality (HR = 9.372, *P* < .001). An increased lactate dehydrogenase value was associated with a low survival (*P* = .002). The international prognostic index score represents a negative risk factor in terms of patient survival (HR = 4.654, *P* < .001). In cases of diffuse large B-cell lymphoma (DLBCL), immunopositivity of *MYD88* is associated with non-germinal center B-cell origin (*P* < .001). The multivariate analysis observed the association between high lactate dehydrogenase value and the immunohistochemical expression of *PIM1* or with the mutant status of the *PIM1* gene representing negative prognostic factors (HR = 2.066, *P* = .042, respectively HR = 3.100, *P* = .004). In conclusion, our preliminary data suggest that the oncogenic mutations of *PIM1* and *MYD88* in our DLBCL cohort may improve the diagnosis and prognosis of DLBCL patients in an advanced stage.

## 1. Introduction

Non-Hodgkin lymphoma (NHL) is a heterogeneous group of tumors that arise from developing lymphocytes and is far more common than Hodgkin lymphoma.^[1,2]^ The most common NHL subtype in South-Eastern Europe was diffuse large B-cell lymphoma (DLBCL) (39%), followed by follicular lymphoma (15,8%) and the relative frequency of DLBCL is significantly higher in South-Eastern Europe compared to Western Europe (WEU) (29,3%) and North America (NA) (28.3%).^[3]^

The family/class of large B-cell lymphomas comprises a spectrum of tumors with varying morphologies, genetic features, and clinical behavior. Diffuse large B-cell lymphoma, not otherwise specified (DLBCL, NOS), represents the largest entity, and is defined by morphology and a mature B-cell phenotype.^[4]^

Diffuse large B-cell lymphoma (DLBCL) is an aggressive non-Hodgkin lymphoma, and despite advances in survival with the introduction of rituximab, about one-third of patients with advanced stage are still unresponsive to current therapy or eventually relapse.^[5,6]^

Most DLBCL, NOS broadly recapitulate the differentiation and maturation mechanisms active in normal B cell development and hence, 2 main subtypes previously defined in the World Health Organization (WHO) 4th edition (revised) continue to be recognized—the germinal center B-cell subtype (GCB) and the activated B-cell subtype (ABC). More recent data from next-generation sequencing studies have illustrated that—despite the use of different sequencing approaches and various clustering algorithms—the genetic landscape of DLBCL, NOS can be used for sub-classification with broad concordance suggesting that the underlying disease biology may indeed be captured by comprehensive genetic landscapes.^[4]^

Lymphoma types/entities are defined by morphological, immunophenotypical, and clinical criteria, but none of these are absolutely specific and defining.^[4]^

The morphological and/or clinical settings defining the entity/type, respectively, overrule the genetic characteristics in such cases. Similarly, an immune deficiency/dysregulation setting overrules other defining parameters (e.g., central nervous system [CNS] localization of immune deficiency/dysregulation-associated DLBCL, Epstein-Barr virus-positive, human immunodeficiency virus [HIV]-positive setting).^[4]^

They are enriched for B-cell antigen receptor pathway mutations such as *in MYD88, CD79B*, provirus integration site for Moloney murine leukemia virus 1 (*PIM1*), and *PRDM1* encoding *BLIMP1*.^[7]^

The myeloid differentiation primary response 88 (*MYD88*) protein is an important adaptor molecule in Toll-like receptors signaling that prompts the activation of NF-kB and the production of both inflammatory cytokines and type I interferons.^[8]^ Previous studies have indicated that *MYD88* mutations in lymphoma are gain-of-function mutations, the most common variant of which is the *L265P* (c.794T>C) mutation in the evolutionarily conserved hydrophobic core of the Toll/IL1 receptor domain of *MYD88*.^[1,9–12]^ Ngo et al found that 29% of ABC DLBCL patients had a leucine (CTG) to proline (CCG) ex-change at position 265 (*L265P*) of the myeloid differentiation main response gene 88, which may be another factor contributing to NF-B overactivity. Therapeutically, inhibiting NF-B signaling may be used to treat DLBCL, which is not compliant.^[5,9]^

The identification as cooperating targets of Proviral Integrations of Moloney virus in murine lymphomas suggested early on that *PIM* serine/threonine kinases play an important role in cancer biology. Elevated levels of *PIM1* were mostly found in hematological malignancies. *PIM1* seems to mediate homing and migration of normal and malignant hematopoietic cells by regulating chemokine receptor surface expression. Knockdown experiments by ribonucleic acid interference or dominant-negative acting mutants suggested that *PIM* kinases are important for the maintenance of a transformed phenotype and therefore potential therapeutic targets.^[13]^

PIM1 expression is correlated with poor prognosis in DLBCL, NOS and the most common *PIM1* mutations identified in patients with poor response to targeted therapy are *G28D, L2V*, and *S97N*.^[14,15]^ Therefore, *PIM1* appears as an attractive target in the therapy of hematopoietic neoplasms and as a biomarker of early progression.

However, the *MYD88 L265P* and *PIM1 p.G28A, p.L184V*, and *p.V197F* mutations have not yet been examined in conjunction with MYD88 and PIM1 protein expression in large B-cell NHL cases.

In this study, we aimed to determine the *MYD88 L265P* and *PIM1 p.G28A, p.L184V, p.V197F* mutation frequency, the level of MYD88 and PIM1 immunohistochemical expression, and their associations with each other and with clinicopathological parameters among patients with large B-cell non-Hodgkin lymphomas in Romania.

Therefore, it may be essential to perform a determination of these mutations and, if necessary, categorize the tumors in light of the existence of the mutation in lymphomas.

## 2. Materials and methods

### 2.1. Patient cohort and tissue tumor specimens

We performed a retrospective study over 10 years (2012–2021) that included cases of mature large B-cell NHL. The cases enrolled in this study were diagnosed in the hematology department of the County Emergency Clinical Hospital of Constanţa and Sacele County Hospital of Brasov. The hospital’s archives and electronic databases were used to extract the data. The patient’s clinical information and evolutionary information came from the hospitalization electronic medical records. The data were evaluated by the attending physician and the hematologist.

Following the surgical procedures, the surgical specimens were sent to the pathology department of the same hospital for examination. First, a gross description was made, along with information about the lesions’ largest diameter, localization, color, and consistency of specimens. The specimens were prepared and processed according to international protocols within the Clinical Service of Anatomic Pathology, County Emergency Hospital, Constanta, and Service of Anatomic Pathology, Sacele Municipal Hospital, Brasov, and by embedding them in paraffin and applying hematoxylin-eosin on the slides. Consecutive sections of formalin-fixed and paraffin-embedded (FFPE) tissue were obtained for deoxyribonucleic acid (DNA) isolation and immunohistochemistry (IHC) analysis.

The final diagnosis was made according to the WHO criteria appropriate to the year in which the patients were diagnosed. Two different pathologists reevaluated the relevant cases while taking into account the most recent WHO classification guidelines (5th edition) and following the guidelines of the International Consensus Classification of Mature Lymphoid Neoplasms.^[4,16]^

All patients were classified according to the Eastern Cooperative Oncology Group (ECOG) Performance Status, Ann Arbor staging system, and International Prognostic Index (IPI) score, following the criteria described in previous studies by Lister et al^[17,18]^

The classification of tumors as GCB-like and as the non-GCB subtype in DLBCL was performed according to the algorithm of Hans et al (2004).^[19]^

The diagnosis of high-grade B-cell lymphoma, not otherwise specified (HGBL, NOS) was made by histological features and immunohistochemical examination.

The scanning of the slides was performed at the Center for Research and Development of the Morphological and Genetic Studies of Malignant Pathology (CEDMOG). The WSIs were obtained using the Huron LE120TM 4000XT scanner (Huron Technolo-gies International Inc., Canada).

Tissue samples were collected from all patients before treatment.

Our study was approved by the Ethics Committee of both hospitals and all the patients signed the informed consent at the time of hospitalization and, conducted in compliance with the Declaration of Helsinki.

### 2.2. Immunohistochemical evaluation

The immunohistochemical evaluation was performed at the Center for Research and Development of the Morphological and Genetic Studies of Malignant Pathology (CEDMOG) using MYD88 (RM306 clone, HIER-DAB method, [NBP2-61565]) and PIM1 (ST0513 clone, HIER-DAB method, [NBP2-67528]) from Novus Biologicals (CO, USA).

Tissue blocks provided by the Clinical Service of Pathology of the Clinical Emergency County Hospital “Sf. Apostol Andrei” and Municipal Hospital of Sacele were cut into 4 µm sections and applied on a slide.

Positive controls, generated by replacing the primary antibody with phosphate-buffered saline, made by using samples of kidney tissue for MYD88 and tonsils tissue for PIM1, that had previously been evaluated, were utilized in each staining run. The number of MYD88- and, PIM1-immunoreactive cells in at least 10 areas (hot spot method) was determined using a 40× objective lens and a light microscope.

The staining protocol followed the producer’s recommendations: for deparaffinized, we used xylene and decreasing grades of alcohol. The Novus Biologicals protocol for PIM1 antibody (ST0513; [NBP2-67528]) and MYD88 antibody (RM306; [NBP2-61565]) immunostaining included HIER in citrate pH 6.0 buffer and incubation overnight at 4 °C with a 1:200 dilution for PIM1 and1:150 for MYD88. The detection stage required a polymer detection kit that included peroxidase, 2.5% normal horse serum, HRP, and DAB reagent—Vector Laboratories, Burlingame, CA. In the final stage, we counterstained with hematoxylin and mounted the glass cover slides.

The evaluation of PIM1 and MYD88 was carried out qualitatively as well as quantitatively. The quality of the expression was assessed as positive or negative, and the amount of PIM1 and MYD88 immunoreactive cells in at least 10 areas was determined using a 40× objective lens and a light microscope.

According to the data presented in a prior study by Choi et al,^[5]^ the following immunohistochemistry scoring system was used to evaluate MYD88 immunohistochemical staining. Scores for staining intensity ranged from 0 (negative), 1 (weak), 2 (moderate), or 3 (intense) and the extent of staining was scored as 0 (0% of tumor area stained), 1 (<10%), 2 (10–50%), or 3 (˃50%). Each example received a total score ranging from 0 to 6 once these 2 scores were added together. The tissue microarray core with the highest score was chosen for statistical analysis in each case. A low and high MYD88 expression, respectively, was determined by the final scores of 2 to 4 and 5 to 6.

### 2.3. DNA isolation

Genomic DNA was extracted using the QIAamp DNA FFPE Tissue Kit (Qiagen, Germany) according to the manufacturer’s protocol. In brief, tissue areas up to 250 mm^2^ and 8 sections with a maximum thickness of 10 mm were used for DNA isolation. Hematoxylin and eosin-stained sections were used as a reference and the largest tumor area (with at least 50% tumor cells) was scraped off with a scalpel under a dissecting microscope. The concentration and purity of DNA samples were measured by Nanodrop One spectrophotometer, where an absorbance ratio A260/A280 = 1.8–2.0, and A260/A230 > 2 was considered acceptable.

### 2.4. Genotyping of the MYD88 (rs387907272, T/C) and PIM1 (rs377274719, G/A; rs200495767 C/G; rs137884665 G/T) polymorphisms

SNPs polymorphisms of the *MYD88* and *PIM1* were identified using a real-time PCR method based on the readymade TaqMan® Genotyping Master Mix and 20× SNP Genotyping Assay (specific for each polymorphism) containing target-specific oligo-nucleotides labeled with a reporter dye at the 5´ end of each probe for distinguishing between the 2 alleles: VIC dye is linked to the 5´ end of the Allele 1 probe and 6FAM dye is linked to the 5´ end of the Allele 2 probe. (Table 1). The DNA concentration was set between 1 to 10 ng per 10 μL of real-time polymerase chain reaction. Shortly, each 10 μL real-time polymerase chain reaction consists of 5 μL TaqMan Genotyping Master Mix (2×), 0.5 μL of TaqMan genotyping assay mix (20×), and a 4.5 μL DNA. Samples were incubated in a 7500 Fast Real-Time System with the following parameters: 95 °C for 10 minutes, 95 °C for 15 seconds, and 60 °C for 1 minute. The last 2 steps of denaturing and annealing/extending were repeated 40 times. Allelic discrimination was made with the help of 7500 Fast Real-Time PCR software, version 2.3.

**Table 1:** SNPs polymorphisms of the MYD88 and PIM1.

Patient number	Rs	Genes	Sequence	VIC/FAM
1	rs387907272	MYD88	CTTGCAGGTGCCCATCAGAAGCGAC[T/C]GATCCCCATCAAGTACAAGGCAATG	T/C
2	rs377274719	PIM1	GTCCCCGTGCTTCCCCCTTTCCTAG[G/A]CAAGGAGAAGGAGCCCCTGGAGTCG	G/A
3	rs200495767	PIM1	CGACCTCAATCGCGGCGAGCTCAAG[C/G]TCATCGACTTCGGGTCGGGGGCGCT	C/G
4	rs137884665	PIM1	GTCGGGGGCGCTGCTCAAGGACACC[G/T]TCTACACGGACTTCGATGGTGAGCC	G/T

### 2.5. Statistical analysis

SPSS Statistics Version 26 (IBM Corporation, NY) was used to analyze statistical data. Both central tendency and variability indicators were applied. For categorical data, Fisher exact test, and for continuous variables, the Mann–Whitney U test and Kruskal–Wallis H test, were used in an analysis of univariate data. To establish the association of data, we used the Pearson correlation coefficient. Survival estimates were performed until March 1, 2023, and they were calculated using the Kaplan–Meier method. Applying the log-rank test, the survival differences between groups were examined. The use of Cox regression analysis allowed for the appreciation of hazard ratios (HRs). Results were considered statistically significant at a *P*-value of <.05.

## 3. Results

### 3.1. Patient’s clinical and demographic characteristics

In the present study, we identified 50 cases of mature large B-cell NHL, these being represented by DLBCL (80%), HGBL (8%), T-cell/histiocyte-rich large B-cell lymphoma (THRLBCL) (8%), and PCLBC (4%). In the case of the cell of origin (COO) of DLBCL, the most common was the GCB 32 of 40 (64%). Mature large B-cell lymphomas affected both sexes equally (M:F—1:1). The average age of the patients was 61.04 years (27–101 years), with the majority of patients being over 60 years old (60%). More than half of the cases were found in the lymph nodes (54%). The other main localizations of large B-cells NHL, according to the analysis of the studied batch, are the gastrointestinal tract (34%) and CNS (8%). Also, the most frequent localization in the GI tract was in the stomach (10%) and the small intestine (10%).

Patients with lymphoma presented a low ECOG performance score (0–1) at admission in most of the cases (70%). According to the patient’s hospitalization medical records, the most frequently reported comorbidities were secondary anemia (74%), hypertension (20%), and diabetes mellitus (14%). The advanced age of the patients, especially those over 60 years, was associated with the presence of high blood pressure (*P* = .038, respectively *P* = .031). Also, an association with the presence of atrial fibrillation was observed in elderly patients (*P* = .016). The presence of dyslipidemia was observed especially in the case of DLBCL 5 of 40 (12,5%), showing significant statistical associations with the female sex (*P* = .018). Hypertension was found in close association with hyperuricemia (*P* = .020), atrial fibrillation (*P* < .001), hematuria (*P* = .002), renal failure (*P* = .003), upper digestive hemorrhage (*P* = .003) and cardiac insufficiency (*P* < .001).

Some of the patients presented antecedents of acute infections in 16% of cases (with *Klebsiella pneumoniae, Staphylococcus epidermidis*, Streptococcus, Bacillus spp., *Pseudomonas aeruginosa; Helicobacter pylori*, and *Candida albicans*) or chronic infections like hepatitis virus C (8%), tuberculosis (8%), hepatitis virus B (4 %) or HIV (4%). The patients with HIV infection were younger than the others without HIV (*P* = .003). A predominance of male patients has been observed in the distribution of infectious disease antecedents (*P* = .007) and the hepatitis virus C was observed mostly in female patients (*P* = .038).

Most of the patients (70%) had a good performance status ECOG (0–1). The main symptoms that led to hospitalization varied, the most common being hyperuricemia (16%), hepatosplenomegaly (16%), or hematuria (12%). The presence of hyperuricemia was associated with the presence of concomitant neoplasms (*P* = .015).

**Figure 1.**
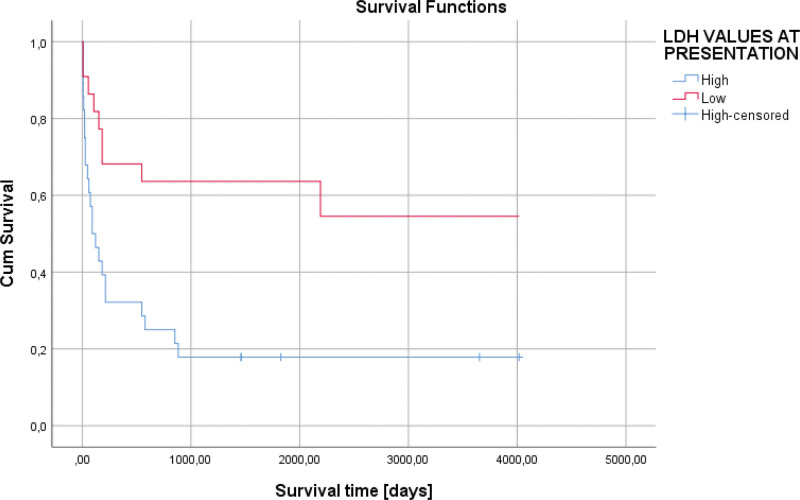
Kaplan–Meier survival graphic that shows a lower survival associated with an increased LDH value at the presentation of the patients (*P* = .002).

**Figure 2.**
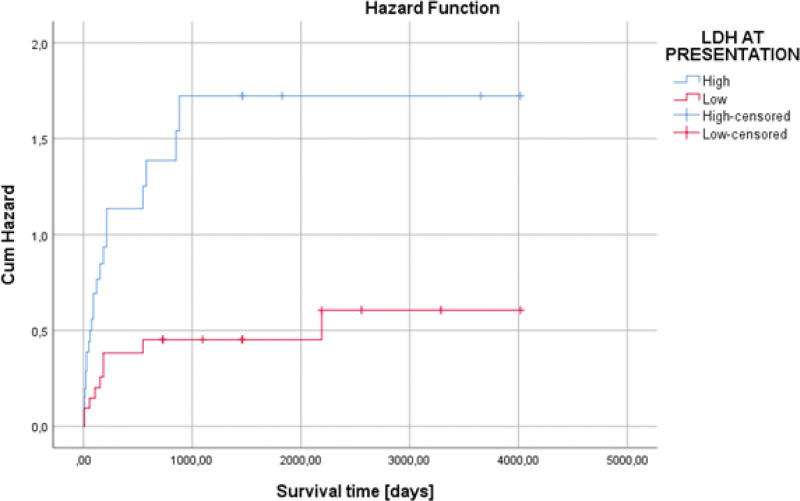
Univariate Cox regression analysis that expresses an elevated LDH value at admission represented an independent negative risk factor in terms of patient survival (HR = 3.100, *P* = .004).

From a biochemical point of view, the most important change was the increase in lactate dehydrogenase (LDH) values (56% of cases). It had an average value of 471.04 IU/L. In the case of anemic syndrome, the average value of hemoglobin was 10.93 g/dl (4.90–15.70 g/dL). Regarding the association between the laboratory tests, we observed an increased LDH at admission that was statistically significantly associated with the presence of anemia (*P* = .033), and also with low hemoglobin values (*P* = .011). Also, we observed a statistically significant association between the LDH values and hemoglobin—the higher the LDH value, the lower the hemoglobin (*P* = .001). However, in the case of increased LDH values, the ECOG performance status was low (0–1), (*P* = .033). In the case of hemoglobin, its decrease was also associated with the presence of upper digestive hemorrhage (*P* = .021), as well as with other concomitant neoplasms (*P* = .046). An increased LDH value was associated with low survival of the patients (*P* = .002), 869.34 days (124.19 weeks) versus 2447.10 days (349.58 weeks), (Fig. 1). An elevated LDH value at admission represented an independent negative risk factor in terms of patient survival (HR = 3.100, *P* = .004) (Fig. 2 and Table 2).

**Table 2:** Univariate and multivariate Cox regression analyses.

	Univariate analysis		Multivariate analysis	
	HR	*P*	HR	*P*
ECOG performance status	9.372	*P* < .001	2.645	*P* = .185
The lack of treatment	6.750	*P* < .001	1.736	*P* = .087
IPI score	4.654	*P* < .001	2.251	*P* = .133
Age ˃ 60	1.460	*P* = .310	7.715	*P* < .001
LDH	1.000	*P* = .108	3.582	*P* = .020
IHC PIM1	1.067	*P* = .856	2.066	*P* = .042
PIM1 mutant	21.056	*P* = .537	3.100	*P* = .004

In most cases, an average percentage of lymphocytes of 2.74% (0.34–22.95%) was observed in the peripheral blood, with a value of 21.53 × 10^3^/L of lymphocytes.

A little over half of the cases had nodal location (54%), being associated with the presence of hepatosplenomegaly (*P* = .039). The most frequent extranodal localizations of the lymphomas were gastrointestinal tract (34%), the majority being represented by the stomach (10%), small bowel (10%), and large bowel (6%). Other frequent localizations were in immune-privileged sites like the brain (8%) testis (2%), or spleen (4%). Patients with lymphoma in the small bowel, spleen, brain, and testis had a high-performance status ECOG (2–4) compared to the other locations (*P* = .001).

**Figure 3.**
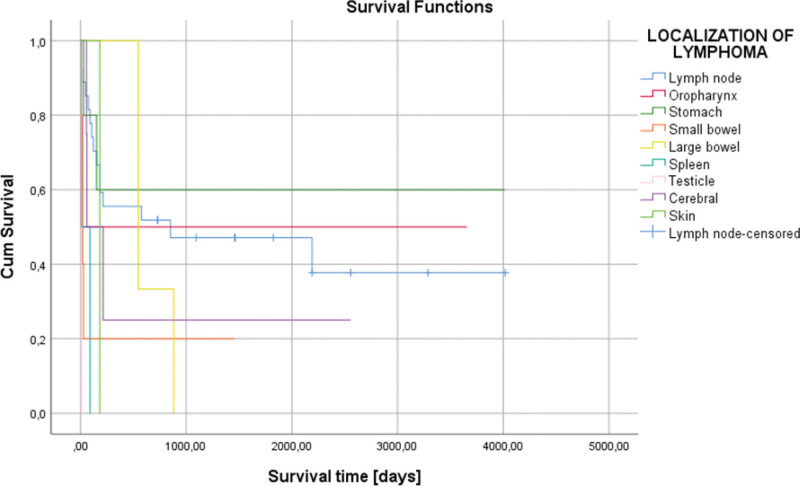
Kaplan–Meier survival graphic that shows a lower survival in the testis and spleen localizations of lymphomas (*P* = .010).

**Figure 4.**
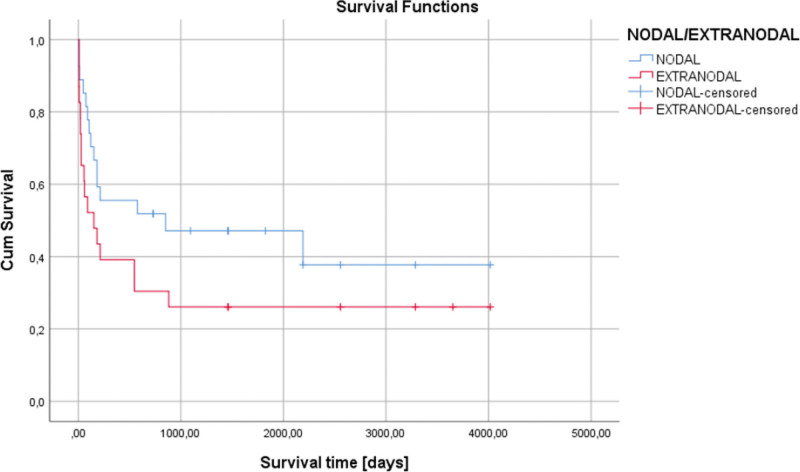
Kaplan–Meier survival graphic that shows a lower survival in the extranodal localizations of lymphomas (*P* = .130).

Depending on the localizations of the lymphoma, the testis or spleen was associated with the lowest survival, 7 days (one week), respectively 49.14 days (7.02 weeks), (Fig. 3). Also, lower survival was observed in the extranodal localization of lymphomas compared with nodal localization of lymphomas (*P* = .130) (Fig. 4).

Ann Arbor staging highlighted a slight majority of cases (52%) in the low-grade category (I–II). The same aspect was found and could be transposed in the international prognostic index score, with 64% of the cases being at low risk. A low-grade Ann Arbor staging was associated with nodal localizations (*P* < .001), and a similar aspect was also noted in the cases with a low IPI score (*P* = .001). A high IPI score was associated with extranodal localizations other than those in the skin or large bowel (*P* < .001). Also, a high IPI score was found in cases of elevated LDH values (*P* = .001).

Unfortunately, not all patients benefited from the treatment, but only 62% of them, and the preferred scheme was rituximab, cyclophosphamide, doxorubicin, vincristine, and prednisone (R-CHOP) (44%). This treatment scheme was especially associated with a low age of the patients (*P* = .034), but also with the nodal localization of the lymphoma (*P* = .004). Both R-CHOP and cyclophosphamide, doxorubicin, vincristine, prednisone (CHOP) protocol schemes were associated with a good performance status ECOG (*P* < .001), with a low Ann Arbor stage (*P* = .005), and with a favorable IPI risk score (*P* < .001).

**Figure 5.**
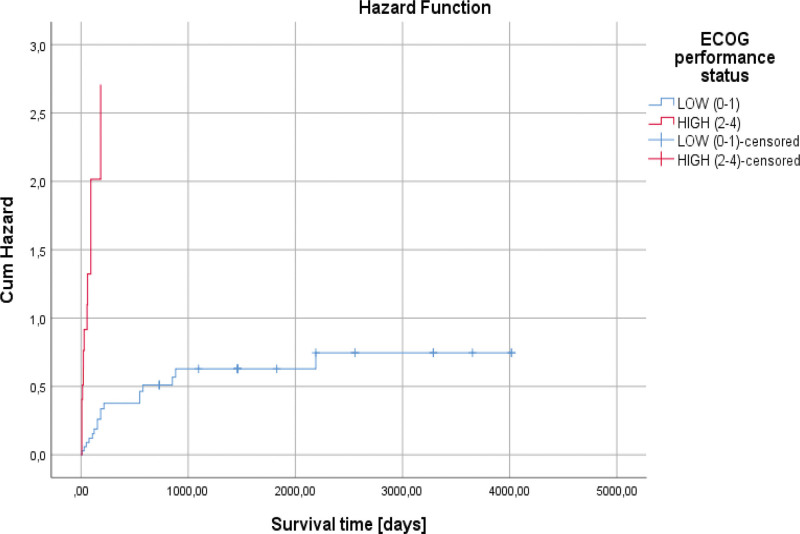
Univariate Cox regression analysis showed that the ECOG performance status represented an independent risk factor predicting mortality (HR = 9.372, *P* < .001).

At the end of this study, only 36% of patients were alive. The average survival was 913.42 days (130.48 weeks). Patients with a good performance status (0–1) had much higher average survival, 2176.49 days (310.92 weeks) vs. 54.36 days (7.76 weeks), compared to those with an altered status (*P* < .001). The ECOG performance status represented an independent risk factor predicting mortality (HR = 9.372, *P* < .001) (Fig. 5 and Table 2).

**Figure 6.**
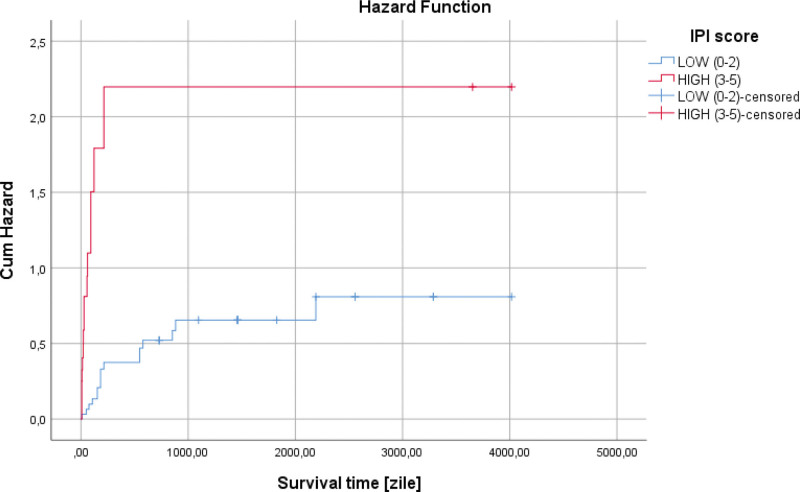
Univariate Cox regression analysis showed that the high risk represented by the IPI score represents a negative risk factor in terms of patient survival (HR = 4.654, *P* < .001).

The most effective treatment for survival was the use of the CHOP scheme, in its case an average survival of 2323.32 days (331.90 weeks) was observed, while in the R-CHOP scheme, an average of 2278.90 days (325.55 weeks) was observed, and those who did not perform chemotherapy treatment had an average of 117.16 days (16.73 weeks), (*P* < .001). The lack of chemotherapeutic treatment represents a risk factor regarding patient mortality (HR = 6.750, *P* < .001) (Table 2). The IPI score was associated with patient survival, so those with high risk had a lower average, 54.36 days (7.76 weeks) versus 2176.49 days (310.92 weeks), compared to those in the low-risk category (*P* < .001). The high risk represented by the IPI score represents a negative risk factor in terms of patient survival (HR = 4.654, *P* < .001) (Fig. 6 and Table 2).

The multivariate analysis of the data shows that the associations between a low-performance status (2–4) with the age of over 60 and an increased LDH with the presence of anemia are risk factors regarding survival (HR = 7.715, *P* < .001, respectively HR = 3.582, *P* = .020) (see Table 2).

The distribution of demographic aspects, tumor characteristics, and personal history are stratified according to diagnosis in Table 3.

**Table 3:** The distribution of demographic aspects, tumor characteristics, and personal history are stratified according to diagnosis.

Clinical features	DLBCL, NOS* (n = 40)	HGBL, NOS† (n = 4)	THRLBCL‡ (n = 4)	PCLBCL-LT§ (n = 2)	*P*-value∥
Age	27–87	47–65	41–77	67–101	
Average	60.78	55.25	58	84	*P* = .287
(>60 years)	62.5%	50%	25%	100%	*P* = .362
Gender					
Male	52.5%	25%	50%	50%	
Female	47.5%	75%	50%	50%	*P* = .921
ECOG¶ status					
Low (0–1)	67.5%	50%	100%	100%	*P* = .412
High (2–4)	32.5%	50%	0%	0%	
Comorbidities					
Secondary anemia	77.5%	75%	25%	100%	*P* = .156
High blood pressure	25%	0%	0%	0%	*P* = .667
Diabetes mellitus	10%	0%	50%	50%	*P* = .066
Dyslipidemia	12.5%	0%	0%	0%	*P* = .456
Atrial fibrillation	12.5%	0%	0%	0%	*P* = .456
Concurrent neoplasms	12.5%	0%	0%	50%	*P* = .335
Cardiac insufficiency	10%	0%	0%	0%	*P* = .636
Gastric ulcer	7.5%	0%	0%	0%	*P* = .669
Antecedent infections	20%	0%	0%	0%	*P* = .291
HVB#	2.5%	25%	0%	0%	*P* = .363
Tuberculosis	7.5%	25%	0%	0%	*P* = .603
HIV**	5%	0%	0%	0%	*P* = .869
HVC††	7.5%	0%	25%	0%	*P* = .603
Presentation					
Hyperuricemia	20%	0%	0%	0%	*P* = .257
Hematuria	10%	50%	0%	0%	*P* = .160
Hepatosplenomegaly	17.5%	25%	0%	0%	*P* = .857
Epilepsy	7.5%	0%	0%	0%	*P* = .669
LDH level					
Normal (< 225)	40%	25%	100%	50%	
High (˃ 225)	60%	75%	0%	50%	*P* = .081
Average	532.28	279.25	174.52	222.50	*P* = .097
Average hemoglobin	10.65	11.23	13.43	11.15	*P* = .184
Lymphocytes (%)	2.06	5.72	7.28	1.27	*P* = .173
Values	21.75	5.43	34.38	23.70	*P* = .003
Tumor location					
Nodal	50%	50%	100%	50%	*P* = .289
Extranodal	50%	50%	50%	50%	
Ann Arbor staging					
Low (I–II)	50%	50%	100%	0%	*P* = .118
High (III–IV)	50%	50%	0%	100%	
IPI score					
Low (0–2)	60%	75%	100%	50%	*P* = .548
High (3–5)	40%	25%	0%	50%	
Treatment					
R-CHOP	45%	0%	75%	50%	*P* = .259
CHOP	17.5%	25%	25%	0%	
Without	37.5%	75%	0%	50%	
Death	65%	75%	25%	100%	*P* = .355
Overall survival (days)	873.25	509.19	2100.13	152.14	*P* = .148

CHOP = cyclophosphamide, doxorubicin, vincristine; prednisone; HGBL, NOS = high-grade B-cell lymphoma, not otherwise specified; IPI score = international prognostic index; LDH = lactate dehydrogenase; R-CHOP = rituximab, cyclophosphamide, doxorubicin, vincristine; prednisone.

* Diffuse large B-cell lymphoma, not otherwise specified.

† High-grade B-cell lymphoma, not otherwise specified.

‡ T-cell/histiocyte-rich large B-cell lymphoma.

§ Primary cutaneous diffuse large B-cell lymphoma, leg type.

∥ *P*-value was assessed with a chi-squared test.

¶ ECOG status—performance status assessment by using Eastern Cooperative Oncology Group.

# HVB—hepatitis virus B.

** Human immunodeficiency virus.

†† Hepatitis virus C.

### 3.2. MYD88 L265P and MYD88 expression

Genetic analysis revealed a mutant status of *MYD88* in 8% of cases and of *PIM1* in 2% of cases (Table 4). *MYD88 L265P* mutation was observed in 4 of 40 (8%) of DLBCL cases.

**Table 4:** Genetic analysis of MYD88 and PIM1: MYD88 L265P, PIM1 p.G28A, p.L184V, p.V197F Mutations Status and MYD88, PIM1 immunohistochemical expressions.

Clinicopathological variables	Clinicopathological variables	MYD88* status (%)				PIM1† status (%)				MYD88 expression (%)				PIM1 expression (%)		
		Mutant	Wild type	*P*-value	Mutant		Wild type	*P*-value	Positive		Negative	*P*-value	Positive		Negative	*P*-value
Gender	Male	25%	52.2%	.609	100%		49%	1	12.5%		57.1%	.049	40.7%		60.9%	.256
	Female	75%	47.8%		0%		51%		87.5%		42.9%		59.3%		39.1%	
Age	< 60 years	25%	41.3%	.641	100%		38.8%	.400	50%		38.1%	.697	40.7%		39.1%	1
	˃ 60 years	75%	58.7%		0%		61.2%		50%		61.9%		59.3%		60.9%	
Tumor location:	Nodal:	50%	54.3%	1	100%		53.1%	1	50%		54.8%	1	59.3%		47.8%	.570
	Extranodal:	50%	45.7%		0%		46.9%		50%		45.2%		40.7%		52.2%	
Large B-cell lymphoma subtype	DLBCL, NOS:	100%	78.3%	1	0%		81.6%	.200	37.5%		88.1%	.003	74.1%		87%	.577
	HGBL, NOS:	0%	8.7%		0%		8.2%		25%		4.8%		11.1%		4.3%	
	THRLBCL:	0%	8.7%		100%		6.1%		12.5%		7.1%		7.4%		8.7%	
	PCLBCL-LT:	0%	4.3%		0%		4.1%		25%		0%		7.4%		0%	
LDH levels	Low < 225:	25%	45.7%	.621	100%		42.9%	.440	62.5%		40.5%	.277	33.3%		56.5%	.153
	High ˃ 225:	75%	54.3%		0%		57.1%		37.5%		59.5%		66.7%		43.5%	
ECOG status	Low (0–1):	25%	73.9%	.075	100%		69.4%	1	87.5%		66.7%	.407	74.1%		65.2%	.548
	High (2–4):	75%	26.1%		0%		30.6%		12.5%		33.3%		25.9%		34.8%	
IPI score	Low (0–2):	50%	65.2%	.612	100%		63.3%	1	75%		61.9%	.694	66.7%		60.9%	.771
	High (3–5):	50%	34.8%		0%		36.7%		25%		38.1%		33.3%		39.1%	
Stage Ann Arbor	Low (I–II):	50%	52.2%	1	100%		51%	1	37.5%		54.8%	.456	55.6%		47.8%	.777
	High (III–IV):	50%	47.8%		0%		49%		62.5%		45.2%		44.4%		52.2%	

HGBL, NOS = high-grade B-cell lymphoma, not otherwise specified.

* The myeloid differentiation primary response 88.

† Proto-Oncogene Protein PIM-1.

Patients with the *L265P* mutation were 1 man and 3 women, with a median age of 68.5 years, ranging from 54 to 82 years. Three patients were older than 60 years. None of the patients with the *L265P* mutation showed a significant association with clinical parameters of DLBCL, including the patient’s age, sex, tumor location, ECOG performance status, LDH level, IPI score, and Ann Arbor stage. All but 2 cases were in the non-GCB subgroup.

**Figure 7.**
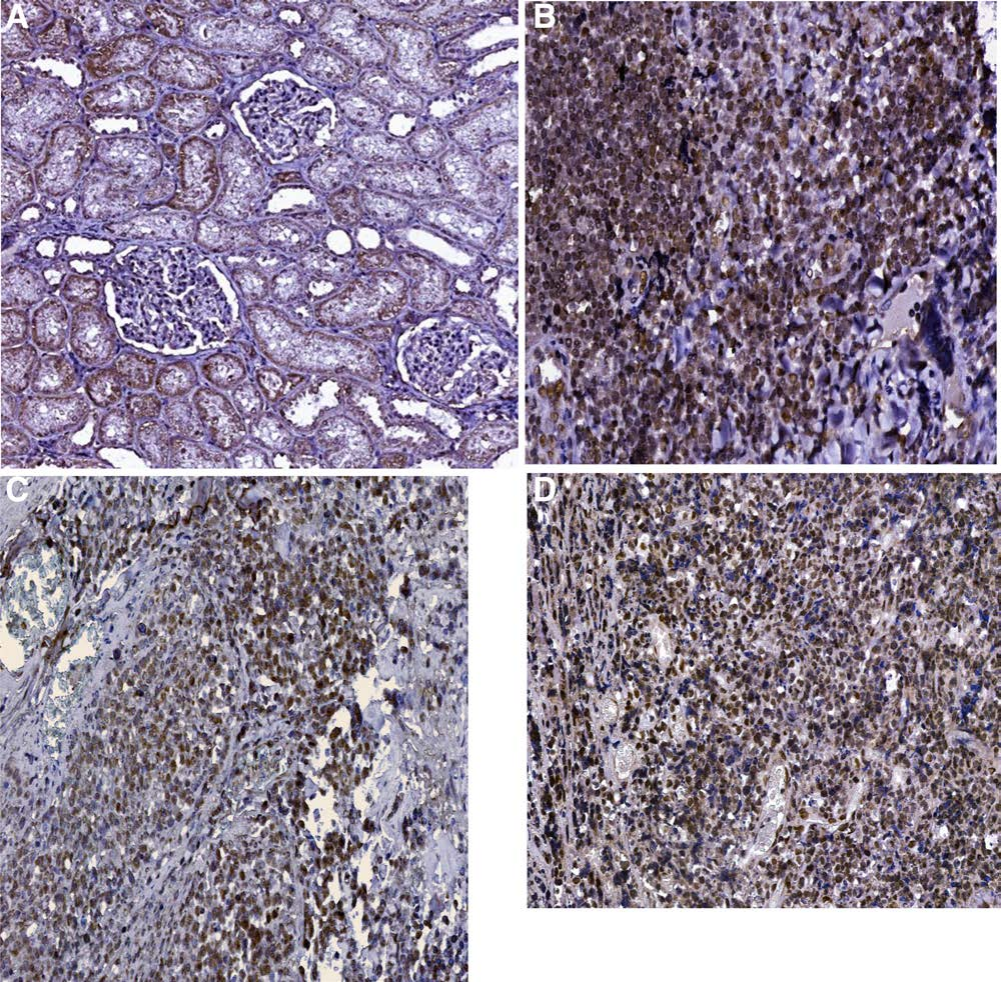
(A–D) Immunohistochemistry evaluation of surgical specimens: (A) the image shows a positive control immunostain using a sample of kidney tissue for MYD88 biomarker (IHC; 40×); (B) the image shows a positive control immunostain using a sample of tonsil tissue for PIM1 biomarker (IHC; 40×); (C) cytoplasmic expression of MYD88 biomarker was positive in scattered cells within the tumor (IHC; 40×); (D) nuclear expression of PIM1 biomarker was positive in most of the cells within the proliferation (IHC; 40×).

MYD88 was expressed in lymphoma cells in 8 (16%) of 50 cases. MYD88 was expressed in the cytoplasm of the lymphoid cells (Fig. 7A and C).

In MYD88 immunopositivity, the quantification of the reaction revealed a low score (2–4) in 10% of cases and a high score (5–6) in 6% of cases. A high score was associated with HGBL lymphomas, while a low score was correlated with THRLBCL and primary cutaneous diffuse large B-cell lymphoma, leg type (PCLBCL-LT) (*P* = .005). In cases of DLBCL, immunopositivity quantified as low and high score is associated with non-GCB origin (*P* < .001), but not with other clinicopathologic parameters.

Three patients died of the disease, and one of them survived and showed a low *MYD88* expression score. Increased survival was observed for wild-type status, 1574.96 days (224.99 weeks), as opposed to mutant status, 695.31 days (99.33 weeks), with no statistically significant significance (*P* = .505).

No statistically significant correlation was observed between MYD88 expression and *L265p MYD88* mutation (Spearman ρ = −0.072, *P* = .617).

### 3.3. PIM1 p.G28A, p.L184V, p.V197F mutations and PIM1 expression

Genetic analysis revealed a mutant status of *PIM1* in 2% of cases (Table 4). *PIM1 p.G28A* was observed in 1 of 4 (25%) THRLBCL cases.

PIM1 was expressed in lymphoma cells in 27 (54%) of 50 cases (Table 4). PIM1 was expressed in the nuclei of the lymphoid cells (Fig. 7B and D).

None of the patients with the *PIM1 p.G28A* mutation showed a significant association with clinical parameters, including the patient’s age, sex, tumor location, ECOG performance status, LDH level, IPI score, and Ann Arbor stage.

The multivariate analysis observed that the association between a high LDH at admission and the immunohistochemical expression of PIM1 or with the mutant status of the *PIM1* gene, represent negative prognostic factors (HR = 2.066, *P* = .042, respectively HR = 3.100, *P* = .004) (see Table 2).

No statistically significant correlation was observed between PIM1 expression and *PIM1 p.G28A* mutation (Spearman ρ = −0.132, *P* = .361). In the case of mutant status, no deaths were observed, these being noted only in the wild-type status.

## 4. Discussion

With an estimated 150,000 new cases each year globally, large B-cell lymphomas account for over 30% of all non-Hodgkin lymphoma cases.^[4,20]^ The updated WHO classification has revised the categorization of large B-cell lymphomas, which are a heterogeneous group of clinicopathological entities that diffuse large B-cell lymphoma, not otherwise specified, is the most common.^[20]^ In the study we performed, DLBCL with NOS was the most prevalent in the group of patients examined (80%), and the findings were in agreement with the data provided by WHO 2022.

The majority of patients with DLBCL, NOS present with nodal disease, and around 30% to 40% of cases present with disease confined to extranodal sites at diagnosis.^[4]^ Virtually any site can be involved but common extranodal sites include the gastrointestinal tract (stomach and ileocecal region).^[4]^ Consistent with those findings, we identified that half of the cases of DLBCL, NOS were found in the lymph nodes 20 of 40 (50%). According to the studies of Bautista-Quach et al, Ghimire P. et al, and Olszewska-Szopa et al, the most frequent site for gastrointestinal NHL is the stomach (60–75% of all cases), followed by the small intestine and the ileocecal region and 2 of the most prevalent diagnoses are diffuse large B-cell lymphoma (DLBCL) and marginal zone lymphoma.^[21–24]^ In our research, the most frequent extranodal localizations of the lymphomas were gastrointestinal tract (34%), the majority being represented by the stomach (10%), small bowel (10%), and large bowel (6%).

The average age of DLBCL at diagnosis, according to Sehn et al, is in the middle of the 60s, and 30% of patients are over 75 years.^[20]^ In our study, the average age of the patients with DLBCL, NOS at diagnosis was similar to the previous estimate (60.78 years).

Limited data are available specifically for HGBL, NOS but at least it is established that the incidence generally increases with age and, consequently, elderly patients are affected most often.^[4]^ In our study, the average age of the patients diagnosed with HGBL, NOS was under those values (55.25 years), underlying the predominance of the disease in younger patients.

THRLBCL accounts for <10% of DLBCL and it preferentially affects middle-aged or older adults (age range 18–90 years), with rare cases affecting children.^[4]^ In our study, the median age of the patients diagnosed with THRLBCL was similar to those values (58 years).

PCLBCL-LT typically occurs in elderly patients with a median age of around 75 years,^[4]^ but in our study, the median age of the patients with PCLBCL-LT was more than those values (84 years).

A definitive diagnosis of DLBCL is necessary according to the WHO classification of Tumors of Hematopoietic and Lymphoid Tissues to obtain data regarding the COO, which may be determined using immunohistochemistry (IHC) stainings or gene expression profiling (GEP).^[4]^ Regarding the molecular subtypes, the frequency of the GCB subtype is around 60%, whereas that of the ABC subtype is between 25% and 30%.^[20]^ Our research identified the cases where the COO could be identified (80%), with a higher predominance of the GCB subtype (64%) and a lower predominance of the non-GCB subtype (16%).

DLBCL, NOS occurs in men slightly more frequently than in women.^[4]^ Similarly, in our case, the male sex diagnosed with DLBCL, NOS is the most affected, observed in 21 of 40 (52.5%) of cases.

In high-grade B-cell lymphoma, NOS cases no sex predilection has been reported. In our research, females are the most affected 3 of 4 (75%).

THRLBCL has a slight male predominance with a male: female ratio, of 1.7–2.6:1^[4]^. In contrast, in our study, THRLBCL affects both sexes equally (M:F = 1:1).

PCLBCL-LT is more common in women, with a female-to-male ratio between 2:1 and 4:1.^[4]^ In contrast, in our study, PCLBCL-LT affects both sexes equally (M:F = 1:1).

R-CHOP therapy is currently the gold standard of care for DLBCL patients across the world.^[25,26]^ However, 20% to 50% of DLBCL patients relapse or are resistant to first-line therapy, making them eligible for second-line and later treatments.^[27,28]^ Unfortunately, there have traditionally been few therapies available once a patient has reached the third line, and overall survival is <30.43 weeks.^[29]^

Our research identified that unfortunately, not all patients benefited from the treatment, but only 62% of them, and the preferred scheme was R-CHOP (44%). In our study, the R-CHOP treatment scheme was especially associated with the low age of the patients (*P* = .034), but also with the nodal localization of the lymphoma (*P* = .004). Both R-CHOP and CHOP protocol schemes were associated with a good performance status ECOG (*P* < .001), with a low Ann Arbor stage (*P* = .005), and with a favorable IPI risk score (*P* < .001). Also, in the current study, the most effective treatment for survival was the use of the CHOP scheme, in its case an average survival of 2323.32 days (331.90 weeks) was observed, while in the R-CHOP scheme, an average of 2278.90 days (325.55 weeks) was observed, and those who did not perform chemotherapy treatment had an average of 117.16 days (16.73 weeks), (*P* < .001). The lack of chemotherapeutic treatment represents a risk factor regarding patient mortality (HR = 6.750, *P* < .001) (see Table 2).

Research studies by Huang et al and Scherer et al have found that LDH is a useful indicator of the size of the tumor in solid and hematological malignancies.^[30,31]^ The LDH value serves as a valuable indicator of the severity of the illness and the efficacy of therapy and is one of the components of the International Prognostic Index (IPI). However, a few studies have shown that in NHL lymphomas, LDH correlates strongly with higher levels of cell-free tumor DNA and might be a surrogate of increased circulating tumor cells.^[31,32]^ The present study demonstrates that in 56% of cases, a value above the normal limit (>225) was observed and it had an average value of 471.04 UI/L. Regarding the association between the laboratory tests, we observed an increased LDH at admission that was statistically significantly associated with the presence of anemia (*P* = .033), and also with low hemoglobin values (*P* = .011). Also, we observed a statistically significant association between the LDH values and hemoglobin—the higher the LDH value, the lower the hemoglobin (*P* = .001). However, in the case of increased LDH values, the ECOG performance status was low (0–1), (*P* = .033).

In our research, regarding the survival of the patients according to this parameter, an increased LDH value was associated with low survival of the patients, 869.34 days (124.19 weeks) versus 2447.10 days (349.58 weeks), (*P* = .002), (Fig. 2). An elevated LDH value at admission represented an independent negative risk factor in terms of patient survival (HR = 3.100, *P* = .004) (Fig. 5 and Table 2).

In a research study by Prochazka et al, higher uric acid levels were linked to lower PFS (hazard ratio [HR]) and overall survival (OS) in univariable time-to-event analysis.^[33]^

Hyperuricemia (20%) was one of the most prevalent comorbidities in our study group, although it had no relationship to a worse OS.

In human ABC DLBCL cell lines, MYD88 forms a complex with the IL-1 receptor-associated kinase 1 and IL-1 receptor-associated kinase 4 and promotes NF-κB and Janus kinase-signal transducer and activator of transcription 3 (JAK-STAT3) signaling, resulting in lymphoma cell survival.^[9]^ After the first report of highly oncogenic *MYD88 L265P* mutation in ABC DLBCL biopsies,^[9]^ the *L265P* mutation was also detected in 5 cases (36%) of 14 primary central nervous system lymphoma (PCNSL) and 11 (69%) of 16 primary cutaneous DLBCL leg-type, respectively.^[9,34,35]^ In our study, genetic analysis revealed a mutant status of *MYD88* in 8% of patients and *L265P* mutation was observed in 4 of 40 (10%) DLBCL cases (Table 4). Also, none of the patients with the *L265P* mutation showed a significant association with clinical parameters of DLBCL, including the patient’s age, sex, tumor location, ECOG performance status, LDH level, IPI score, and Ann Arbor stage.

Patients with GCB DLBCL demonstrated a lowered mutation frequency, whereas patients with non-GCB DLBCL exhibited a mutant status of *MYD88* in 27.8% of cases. These results are consistent with other studies’ findings that the prevalence of *MYD88 L265P* mutations in DLBCL patients ranged from 6.5% to 19%.^[5,9,36,37]^ In our study, the mutation frequency was similar between GCB and non-GCB DLBCL groups.

Overall, the subtypes of DLBCL still represent a heterogeneous group of neoplasms when the presence of *MYD88 L265P* mutation is taken into account.^[1]^

Sanger sequencing further confirmed the presence of *MYD88 L265P* in patients with mature B-cell NHL. Depending on the laboratory circumstances, both analytical approaches might be employed in clinical testing situations because the results were the same as those obtained by sequencing analysis. However, it should be highlighted that in FFPE samples with fragmented nucleic acids, Sanger sequencing may not be able to detect lower-frequency mutations and may result in inadequate PCR amplification templates.^[1]^

Of the 4 *MYD88 L265P* mutations, 2 had nodal involvement, while the other 2 had extranodal involvement, including immune-privileged sites (brain). The patients with extranodal involvement were in the advanced stage (Ann Arbor stage III-IV). Since the *MYD88* mutation is present in several B-cell lymphomas, it has been hypothesized that the *MYD88* mutation represents an early molecular event of lymphomagenesis.^[38]^ A study by Fujiishi et al did not detect the mutation in a diagnostic biopsy specimen of a patient with DLBCL, although the postmortem sample of a patient with DLBCL tested positive for the mutation. They hypothesized that the mutation could be linked to a more aggressive phenotype.^[39]^ According to our findings, individuals with the *MYD88 L265P* mutation were characterized by being at an advanced stage, which raises the possibility that the mutation may be crucial in the development of lymphoma and be linked to a worse prognosis for the disease.

According to a study by Kraan et al, *MYD88* mutations were relatively uncommon in activated B-cell-like (ABC) DLBCLs arising from the lymph nodes or intestine; however, the mutation rate was higher in tumors originating from immune-privileged sites.^[40]^ Also, other studies have indicated that *MYD88* mutations were more prevalent in lymphomas of the primary central nervous system.^[34,41]^

In contrast, in our research the *MYD88 L265P* mutation was detected in half of the patients with non-GCB DLBCL with nodal involvement, while the remaining mutation was present in an immune-privileged site: the CNS (brain). Similarly, only half of the patients with GCB DLBCL with nodal involvement showed *MYD88 L265P* mutation.

As far as we are aware, only Choi et al and Caner et al evaluated MYD88 expression by IHC analysis in DLBCL. The study of Choi et al did not identify any associations between MYD88 expression and clinicopathological variables such as stage or IPI score.^[5]^ In contrast, the study of Caner et al found a reverse relationship between MYD88 overexpression and stage and IPI score.^[1]^ In our research, we also analyzed MYD88 protein expression by IHC analysis in mature B-cell NHLs, and similar to the results reported by Choi et al and by Caner et al, our study failed to reveal the correlation between *MYD88* mutation and expression. A potential limitation of our study is that we focused solely on *MYD88 L265P*, the most prevalent mutation found in lymphomas; other *MYD88* variants, such as *S222R* and *T294P*, could be responsible for the altered protein expression. Therefore, more research is required to understand other genetic and epigenetic changes that underlie the MYD88 expression found in several B-cell NHL patients.

According to studies by Coiffier et al and Fu et al, R-CHOP–treated patients with DLBCL are known to have better survival than CHOP-treated patients.^[6,42]^

In other studies by Ruiz-Delgado et al and Winter et al, the group treated with rituximab plus cyclophosphamide, doxorubicin, vincristine, and prednisone (R-CHOP), and the CHOP-treated group showed no significant difference in disease-free survival and OS.^[43,44]^ In contrast, in our cohort, the group of patients treated with CHOP had better survival than R-CHOP-treated patients, 2323.32 days (331.90 weeks) versus 2278.90 days (325.55 weeks), but similar to other studies, showed no significant difference in OS.

According to a study by Pasqualucci et al, they have reported that an aberrant hypermutation activity targets multiple loci, including the proto-oncogenes *PIM1, MYC, RhoH/TTF (ARHH*), and *PAX5*, in more than 50% of diffuse large-cell lymphomas, which are tumors derived from germinal centers.^[45]^ Overall, the results indicate that the hypermutation of *PIM1, MYC, RhoH/TTF*, and *PAX5* is not common to all germinal-center-derived tumors, but is instead largely restricted to DLBCL and alter-natively, different mutated genes may be selected in different lymphoma types.^[45]^

In another study by Zhou et al, the most frequently mutated genes in their patient cohort diagnosed with non-Hodgkin lymphoma were *PIM1* (77.27%), *MYD88* (63.64%), *CD79B* (59.09%), and *KMT2D* (50.0%).^[15]^ In our study, *PIM1 p.G28A* mutation was observed in 2% of NHL cases and 1 of 4 (25%) THRLBCL cases.

According to Zhou et al, PIM1 and MYD88 were highly expressed in the patient’s cohort and were related to their OS time, and the high expression of PIM1 or MYD88 was correlated with a higher risk score, and the high expression of MYD88 was also correlated with the elevated level of LDH. However, there was no significant correlation with patient age, gender, and/or type.^[15]^ Also, the same research report has shown that multivariate Cox regression model analysis including risk score, LDH level, treatment method, and PIM1 and MYD88 expression status indicated that the expression status of MYD88 was an independent predictor of OS with a HR of 0.004.^[15]^ Consistent with those studies, in our case multivariate analysis observed that the association between a high LDH at admission and the immunohistochemical expression of PIM1 or with the mutant status of the *PIM1* gene represents negative prognostic factors (HR = 2.066, *P* = .042, respectively HR = 3.100, *P* = .004) (see Table 2).

## 5. Conclusions

In conclusion, our study shows that, compared to previously reported studies, the incidence of *MYD88 L265P* and *PIM1 p.G28A* mutations in patients with DLBCL is lower and give a clear picture of the mutational landscape in DLBCL, which may lead to novel ideas for treating the condition.

Thus, regardless of the *MYD88 L265P* mutation, MYD88 expression may have significant implications on the progression of DLBCL, independent of *MYD88 L265P* mutation.

Because of this, we suggest that MYD88 expression and the L265P mutation should only be utilized as prognostic indicators for instances of advanced-stage diseases. Independent of the mutation, MYD88 expression does not indicate the prognosis of the illness.

Regarding PIM1, because of the association between a high LDH at admission and the immunohistochemical expression of PIM1 or with the mutant status of the *PIM1* gene, we hypothesized that both immunohistochemical and expression of PIM1 could be used as prognostic factors in patients diagnosed with large B-cell lymphomas.

## Acknowledgments

This research was performed in the Center for Research and Development of the Morphological and Genetic Studies of Malignant Pathology from the “Ovidius” University of Constanţa.

This work is supported by the project PROINVENT in the framework of the Human Resources Development Operational program 2014–2020, financed from the European Social Fund under contract number 62487/03.06.2022 POCU 993/6/13/–Cod SMIS: 153299.

We would like to thank our colleague Costel Stelian Brinzan for being involved in this project and for providing an interpretation of the results. Also, we are grateful to all the participants, doctors, and researchers involved in this project.

## Author contributions

**Conceptualization:** Miruna Cristian, Mariana Așchie, Anca-Antonela Nicolau, Ionuț Poinăreanu.

**Data curation:** Miruna Cristian, Mariana Așchie, Anca-Florentina Mitroi, Cristian-Ionuț Orășanu.

**Formal analysis:** Anca-Antonela Nicolau, Cristian-Ionuț Orășanu.

**Funding acquisition:** Miruna Cristian, Mariana Așchie.

**Investigation:** Miruna Cristian, Cristian-Ionuț Orășanu.

**Methodology:** Miruna Cristian, Mariana Deacu, Mădălina Boșoteanu, Gabriela-Izabela Bălțătescu, Cristian-Ionuț Orășanu.

**Project administration:** Miruna Cristian, Mariana Așchie, Andreea-Daniela Caloian.

**Resources:** Miruna Cristian, Anca-Florentina Mitroi, Mariana Deacu, Mădălina Boșoteanu, Ana-Maria Crețu, Andreea-Daniela Caloian, Ionuț Poinăreanu.

**Software:** Miruna Cristian, Gabriela-Izabela Bălțătescu.

**Supervision:** Miruna Cristian, Mariana Așchie.

**Validation:** Miruna Cristian, Mariana Așchie, Mădălina Boșoteanu, Andreea-Georgiana Stoica, Anca-Antonela Nicolau, Manuela Enciu, Ionuț Poinăreanu.

**Visualization:** Miruna Cristian, Gabriela-Izabela Bălțătescu.

**Writing – original draft:** Miruna Cristian, Mariana Așchie, Anca-Florentina Mitroi, Mariana Deacu, Cristian-Ionuț Orășanu, Ionuț Poinăreanu.

**Writing – review & editing:** Anca-Florentina Mitroi, Mădălina Boșoteanu, Andreea-Georgiana Stoica, Anca-Antonela Nicolau, Manuela Enciu, Cristian Ionuț Orășanu, Ionuț Poinăreanu.

## References

[1] Caner V Sen Turk N Baris IC. MYD88 expression and L265P mutation in mature B-cell non-Hodgkin lymphomas. Genet Test Mol Biomarkers. 2015;19:372–8. 25978699 10.1089/gtmb.2015.0041

[2] Aschie M Stoica AG Mitroi AF. Synchronous association of two types of indolent lymphomas. Rev Chim. 2018;69:3653–5.

[3] Dotlic S Perry AM Petrusevska G. Classification of non-Hodgkin lymphoma in South-eastern Europe: review of 632 cases from the international non-Hodgkin lymphoma classification project. Br J Haematol. 2015;171:366–72. 26213902 10.1111/bjh.13586

[4] Coupland SE Ott G Siebert R. B-cell lymphoid proliferations and lymphomas. In: WHO Classification of Tumours Editorial Board. Haematolymphoid tumours [Internet; beta version ahead of print]. Lyon (France): International Agency for Research on Cancer; 2022 [cited 2023 May 21]. (WHO Classification of tumours series, 5th ed.; vol. 11). Available from: https://tumourclassification.iarc.who.int/chapters/63.

[5] Choi J-W Kim Y Lee J-H. MYD88 expression and L265P mutation in diffuse large B-cell lymphoma. Hum Pathol. 2013;44:1375–81. 23380077 10.1016/j.humpath.2012.10.026

[6] Coiffier B Thieblemont C Van Den Neste E. Long-term outcome of patients in the LNH-98.5 trial, the first randomized study comparing rituximab-CHOP to standard CHOP chemotherapy in DLBCL patients: a study by the Groupe d’Etudes des Lymphomes de l’Adulte. Blood. 2010;116:2040–5. 20548096 10.1182/blood-2010-03-276246 PMC2951853

[7] Pasqualucci L Dalla-Favera R. The genetic landscape of diffuse large B-cell lymphoma. Semin Hematol. 2015;52:67–76. 25805586 10.1053/j.seminhematol.2015.01.005 PMC4646421

[8] O’Neill LA Golenbock D Bowie AG. The history of Toll-like receptors – redefining innate immunity. Nat Rev Immunol. 2013;13:453–60. 23681101 10.1038/nri3446

[9] Ngo VN Young RM Schmitz R. Oncogenically active MYD88 mutations in human lymphoma. Nature. 2011;470:115–9. 21179087 10.1038/nature09671 PMC5024568

[10] Treon SP Xu L Yang G. MYD88 L265P somatic mutation in Waldenström’s macroglobulinemia. N Engl J Med. 2012;367:826–33. 22931316 10.1056/NEJMoa1200710

[11] Martínez-Trillos A Pinyol M Navarro A. Mutations in TLR/MYD88 pathway identify a subset of young chronic lymphocytic leukemia patients with favorable outcome. Blood. 2014;123:3790–6. 24782504 10.1182/blood-2013-12-543306

[12] Varettoni M Arcaini L Zibellini S. Prevalence and clinical significance of the MYD88 (L265P) somatic mutation in Waldenstrom’s macroglobulinemia and related lymphoid neoplasms. Blood. 2013;121:2522–8. 23355535 10.1182/blood-2012-09-457101

[13] Brault L Gasser C Bracher F. PIM serine/threonine kinases in the pathogenesis and therapy of hematologic malignancies and solid cancers. Haematologica. 2010;95:1004–15. 20145274 10.3324/haematol.2009.017079 PMC2878801

[14] Fukumura K Kawazu M Kojima S. Genomic characterization of primary central nervous system lymphoma. Acta Neuropathol. 2016;131:865–75. 26757737 10.1007/s00401-016-1536-2

[15] Zhou Y Liu W Xu Z. Analysis of genomic alteration in primary central nervous system lymphoma and the expression of some related genes. Neoplasia. 2018;20:1059–69. 30227305 10.1016/j.neo.2018.08.012 PMC6141698

[16] Campo E Jaffe ES Cook JR. The International Consensus Classification of Mature Lymphoid Neoplasms: a report from the Clinical Advisory Committee. Blood. 2022;140:1229–53. 35653592 10.1182/blood.2022015851 PMC9479027

[17] Lister TA Crowther D Sutcliffe SB. Report of a committee convened to discuss the evaluation and staging of patients with Hodgkin’s disease: Cotswolds meeting. J Clin Oncol. 1989;7:1630–6. 2809679 10.1200/JCO.1989.7.11.1630

[18] International Non-Hodgkin’s Lymphoma Prognostic Factors Project. A predictive model for aggressive non-Hodgkin’s lymphoma. N Engl J Med. 1993;329:987–94. 8141877 10.1056/NEJM199309303291402

[19] Hans CP Weisenburger DD Greiner TC. Confirmation of the molecular classification of diffuse large B-cell lymphoma by immunohistochemistry using a tissue microarray. Blood. 2004;103:275–82. 14504078 10.1182/blood-2003-05-1545

[20] Sehn LH Salles G. Diffuse Large B-Cell Lymphoma. N Engl J Med. 2021;384:842–58. 33657296 10.1056/NEJMra2027612 PMC8377611

[21] Bautista-Quach MA Ake CD Chen M. Gastrointestinal lymphomas: Morphology, immunophenotype, and molecular features. J Gastrointest Oncol. 2012;3:209–25. 22943012 10.3978/j.issn.2078-6891.2012.024 PMC3418529

[22] Boşoteanu C Boşoteanu M Aşchie M. Mădălina Boşoteanu, and Mariana Aşchie. “Differential diagnosis issues in a case of gastric carcinoma associated with leukemoid reaction.”. Rom J Morphol Embryol. 2009;50:701–5. 19942969

[23] Ghimire P Wu GY Zhu L. Primary gastrointestinal lymphoma. World J Gastroenterol. 2011;17:697–707. 21390139 10.3748/wjg.v17.i6.697 PMC3042647

[24] Olszewska-Szopa M Wróbel T. Gastrointestinal non-Hodgkin lymphomas. Adv Clin Exp Med. 2019;28:1119–24. 31414733 10.17219/acem/94068

[25] Gena K Wenzhen G Ruben GWQ. Epidemiology of diffuse large B-cell lymphoma (DLBCL) and follicular lymphoma (FL) in the United States and Western Europe: population-level projections for 2020–2025. Leuk Lymphoma 2022;63:54–63. 34510995 10.1080/10428194.2021.1975188

[26] Tilly H Gomes da Silva M Vitolo U. ESMO Clinical Practice Guidelines for diagnosis, treatment and follow-up. Ann Oncol. 2015;26(Suppl 5):v116–25. 26314773 10.1093/annonc/mdv304

[27] Purdum A Tieu R Reddy SR. Direct costs associated with relapsed diffuse large B-cell lymphoma thera-pies. Oncologist. 2019;24:1229–36. 30850561 10.1634/theoncologist.2018-0490 PMC6738309

[28] Dreyling M Ghielmini M Rule S. Newly diagnosed and relapsed follicular lymphoma: ESMO Clinical Practice Guidelines for diagnosis, treatment and follow-up. Ann Oncol. 2021;32:298–308. 33249059 10.1016/j.annonc.2020.11.008

[29] Halwani AS Chien HC Morreall DK. Survival patterns in patients with relapsed or refractory diffuse large B cell lymphoma: treatment trajectories and responses after the first relapse. Blood. 2019;134:1622.

[30] Huang AC Postow MA Orlowski RJ. T-cell invigoration to tumour burden ratio associated with anti-PD-1 response. Nature. 2017;545:60–5. 28397821 10.1038/nature22079 PMC5554367

[31] Scherer F Kurtz DM Newman AM. Distinct biological subtypes and patterns of genome evolution in lymphoma revealed by circulating tumor DNA. Sci Transl Med. 2016;8:364ra–155. 10.1126/scitranslmed.aai8545 PMC5490494 27831904

[32] Kurtz DM Green MR Bratman SV. Noninvasive monitoring of diffuse large B-cell lymphoma by immunoglobulin high-throughput sequencing. Blood. 2015;125:3679–87. 25887775 10.1182/blood-2015-03-635169 PMC4463733

[33] Prochazka KT Melchardt T Posch F. NCCN-IPI score-independent prognostic potential of pretreatment uric acid levels for clinical outcome of diffuse large B-cell lymphoma patients. Br J Cancer. 2016;115:1264–72. 27764838 10.1038/bjc.2016.325 PMC5104895

[34] Montesinos-Rongen M Godlewska E Brunn A. Activating L265P mutations of the MYD88 gene are common in primary central nervous system lymphoma. Acta Neuropathol. 2011;122:791–2. 22020631 10.1007/s00401-011-0891-2

[35] Pham-Ledard A Cappellen D Martinez F. MYD88 somatic mutation is a genetic feature of primary cutaneous diffuse large B-cell lymphoma, leg type. J Invest Dermatol. 2012;132:2118–20. 22495176 10.1038/jid.2012.102

[36] Lohr JG Stojanov P Lawrence MS. Discovery and prioritization of somatic mutations in diffuse large B-cell lymphoma (DLBCL) by whole-exome sequencing. Proc Natl Acad Sci U S A. 2012;109:3879–84. 22343534 10.1073/pnas.1121343109 PMC3309757

[37] Bohers E Mareschal S Bouzelfen A. Targetable activating mutations are very frequent in GCB and ABC diffuse large B-cell lymphoma. Genes Chromosomes Cancer. 2014;53:144–53. 24327543 10.1002/gcc.22126

[38] Landau DA Carter SL Stojanov P. Evolution and impact of subclonal mutations in chronic lymphocytic leukemia. Cell. 2013;152:714–26. 23415222 10.1016/j.cell.2013.01.019 PMC3575604

[39] Fujiishi K Kitazawa R Nagai Y. Acquisition of MYD88 L265P mutation during treatment of diffuse large B cell lymphoma of the parotid gland. Virchows Arch. 2014;464:121–4. 24259032 10.1007/s00428-013-1514-1

[40] Kraan W Horlings HM van Keimpema M. High prevalence of oncogenic MYD88 and CD79B mutations in diffuse large B-cell lymphomas presenting at immune-privileged sites. Blood Cancer J. 2013;3:e139. 24013661 10.1038/bcj.2013.28 PMC3789201

[41] Gonzalez-Aguilar A Idbaih A Boisselier B. Recurrent mutations of MYD88 and TBL1XR1 in primary central nervous system lymphomas. Clin Cancer Res. 2012;18:5203–11. 22837180 10.1158/1078-0432.CCR-12-0845

[42] Fu K Weisenburger DD Choi WW. Addition of rituximab to standard chemotherapy improves the survival of both the germinal center B-cell-like and non-germinal center B-cell-like subtypes of diffuse large B-cell lymphoma. J Clin Oncol. 2008;26:4587–94. 18662967 10.1200/JCO.2007.15.9277

[43] Ruiz-Delgado GJ Gómez-Almaguer D Tarín-Arzaga LC. Is there a benefit to adding rituximab to CHOP in the overall survival of patients with B-cell non-Hodgkin’s lymphoma in a developing country? Hematology. 2012;17:193–7. 22889514 10.1179/1607845412Y.0000000006

[44] Winter JN Weller EA Horning SJ. Prognostic significance of Bcl-6 protein expression in DLBCL treated with CHOP or R-CHOP: a prospective correlative study. Blood. 2006;107:4207–13. 16449523 10.1182/blood-2005-10-4222 PMC1895783

[45] Pasqualucci L Neumeister P Goossens T. Hypermutation of multiple proto-oncogenes in B-cell diffuse large-cell lymphomas. Nature. 2001;412:341–6. 11460166 10.1038/35085588

